# Mind the Gap: Social Media Engagement by Public Health Researchers

**DOI:** 10.2196/jmir.2982

**Published:** 2014-01-14

**Authors:** Brett Keller, Alain Labrique, Kriti M Jain, Andrew Pekosz, Orin Levine

**Affiliations:** ^1^Johns Hopkins School of Public HealthBaltimore, MDUnited States; ^2^Johns Hopkins School of Public HealthDepartment of International Health and Department of EpidemiologyBaltimore, MDUnited States; ^3^Johns Hopkins School of Public HealthDepartment of Molecular Microbiology and Immunology and Department of Environmental Health SciencesBaltimore, MDUnited States; ^4^Bill & Melinda Gates FoundationSeattle, WAUnited States

**Keywords:** Internet, social media, public health, blogging

## Abstract

**Background:**

The traditional vertical system of sharing information from sources of scientific authority passed down to the public through local health authorities and clinicians risks being made obsolete by emerging technologies that facilitate rapid horizontal information sharing. The rise of Public Health 2.0 requires professional acknowledgment that a new and substantive forum of public discourse about public health exists on social media, such as forums, blogs, Facebook, and Twitter.

**Objective:**

Some public health professionals have used social media in innovative ways: to surveil populations, gauge public opinion, disseminate health information, and promote mutually beneficial interactions between public health professionals and the lay public. Although innovation is on the rise, most in the public health establishment remain skeptical of this rapidly evolving landscape or are unclear about how it could be used. We sought to evaluate the extent to which public health professionals are engaged in these spaces.

**Methods:**

We conducted a survey of professorial- and scientist-track faculty at the Johns Hopkins Bloomberg School of Public Health in Baltimore, Maryland, USA. We asked all available faculty via email to complete a 30-question survey about respondent characteristics, beliefs about social media, and usage of specific technologies, including blogs, Facebook, Twitter, and YouTube.

**Results:**

A total of 181 (19.8%) of 912 professor- and scientist-track faculty provided usable responses. The majority of respondents rarely used major social media platforms. Of these 181 respondents, 97 (53.6%) had used YouTube, 84 (46.4%) had used Facebook, 55 (30.4%) had read blogs, and 12 (6.6%) had used Twitter in the prior month. More recent degree completion was the best predictor of higher usage of social media. In all, 122 (67.4%) agreed that social media is important for disseminating information, whereas only 55 (30.4%) agreed that social media is useful for their research. In all, 43 (23.8%) said social media was helpful for professional career advancement, whereas 72 (39.8%) said it was not. Only 43 (23.8%) faculty said they would employ a full- or part-time social media consultant, and 30 (16.6%) currently employed one.

**Conclusions:**

Despite near-universal appreciation of the potential for social media to serve as a component of public health strategy, a small minority are actually engaged in this space professionally, whereas most are either disinterested or actively opposed to professional engagement. Social media is seen by most as more useful for spreading information than obtaining it. As public discourse on a number of critical health topics continues to be influenced and sometimes shaped by discussions online from Twitter to Facebook, it would seem that greater discourse is needed about when and how public health professionals should engage in these media, and also how personal, institutional, and professional barriers to greater use of social media may be overcome.

## Introduction

### Background

Over the past 2 decades, the Internet has become an important source of public information. Recently, with the growth and global penetration of social networks, a wide range of online platforms have become important forums for public dialog about health and health care. Across the globe, we posit that a sea change is occurring, characterized not by seekers of static reference information from Internet sources, but by those looking to engage in interactive, bidirectional communication with global communities of like individuals sharing common health aspirations or challenges. As proposed by Evgeny Morozov [1] in his recent book *To Save Everything, Click Here*, we find ourselves rapidly ascending to dizzying heights of “technologic solutionism,” characterized by the devolution of trust in traditional sources of public health information and a growing reliance on the “wisdom of crowds and the marketplace of ideas.” Social media has taken on a new and important role in public discourse and debate, ranging from the mundane to issues of public health significance. Social media platforms, Web- or mobile-based, facilitate interaction by letting users create, share, and view user-generated content. Users can be transformed from passive consumers into content producers [2].

Even before the rise in popularity of social media, patients were increasingly accustomed to seeking medical information online. In a survey of patients from a primary care internal medicine private practice, 53.5% of respondents said they used the Internet to find medical information [3]. A further 60% of those who used the Internet to find medical information believed the quality of information that they found online was the same as or better than what their doctors provided [3]. A 2013 online survey confirmed that health professionals and patients use social media for different purposes, with patients seeking knowledge, garnering social support, and exchanging advice, whereas health professionals communicate with colleagues and market their services [4].

In the past decade, however, many reviews of the quality of medical information available on the Internet based on reviews by clinical experts have rated information as poor and often potentially dangerous [5]. A 2007 systematic review of online information about inflammatory bowel disease identified many sources, but these were characterized by highly variable quality [6]. Eysenbach [7], in his 1998 *BMJ* review, proposed that although the Internet’s inherent “anarchic nature” was essential for uncensored debate, this same attribute can allow poor quality and even dangerous misinformation to proliferate.

When the interactive element of social media is added to the ease of finding information (and potential misinformation) online, the speed and reach of public health debate is often unprecedented, especially in areas that are fraught with controversy. The tools required to create sophisticated online content are widely accessible and information that seems to be authoritative or scientifically valid can be generated at low cost. The result may be that distinguishing science from opinion is much more challenging today for the lay public than it may have been a decade ago. Civil society organizations ranging from patient and special interest groups to so-called “citizen advocacy” groups are increasingly driving public discussion around the introduction of new vaccines [8]. Social media is being explored as a means to deliver some interventions, although the full potential of these strategies remain to be demonstrated [9,10]. The capacity of social networking and social media technologies to effect sea changes in society were made evident by the events of the 2011 “Arab Spring,” demonstrating how ideas and information could spread virally across population [11].

Still, a strong health professional view persists that the user-generated content of social media sites are little more than backchannels, which serve mainly to spread “misinformation and rumor” [3]. This divide is further illustrated by recent findings that patients are more likely than physicians to use social media sites to access or discuss health information [12]. A review of social media usage by local health departments found that communication has been mostly one-way, from departments to the public, but that dialog and engagement are increasing [13].

We argue that social media can be seen as a new landscape for dialog and public health insight, where researchers can gather health information, disseminate research findings, and provide guidance. Opportunities for research abound because social media users are surprisingly open in discussing their own health [14]. Patient-centered online communities, such as PatientsLikeMe.com, have been used to gain insight into rare disease conditions, patient-reported drug-related side effects, and even to validate new instruments [15-17]. Researchers have used sophisticated natural language processing and big data tools to analyze conversations on Twitter, and have been able to predict annual influenza and other epidemic diseases with remarkable accuracy [14]. Scanfield and colleagues [18] used Twitter in 2010 as a means of exploring antibiotic misuse and sharing. As early as 2008, Collier and colleagues [19] had developed Web-based text mining systems to identify and map “public health rumors” into a system they coined BioCaster. These examples illustrate how social media sites, ranging from Twitter to Facebook and even the media-sharing site YouTube, offer novel platforms for health information exchange. Despite initial skepticism about the reach or impact of these platforms, it has become clear that these networks continue to grow, and younger populations are likely to increase their reliance on these sources for public health and medical guidance [20].

### Objective

Although social media sites seem to be increasingly important tools for personal health information exchange, relatively few empirical studies have examined characteristics of those who use social media or the potential health effects of accessing user-generated content [21]. There is even less published information about how and why public health researchers and practitioners use social media professionally. As the public discourse continues to mature within these virtual spaces, it will be critical to identify opportunities to engage health professionals in the dialog. Although it may be overly ambitious to seek to balance this conversation, further study may identify ways to improve the dissemination of valid information and influence positive behavior change [18].

We sought to illustrate this potential by evaluating the extent to which public health researchers are engaged in these spaces. To that end, we conducted a survey of professorial- and scientist-track faculty at the Johns Hopkins Bloomberg School of Public Health, the oldest and largest school of public health in the United States, to begin filling in this gap in public health knowledge. We investigated Johns Hopkins faculty beliefs about social media and their use of various social media tools. A follow-up survey is planned in 2014 to track changes in opinions and activities over time.

## Methods

In April 2011, we conducted an online survey of social media beliefs and practices among faculty at the Johns Hopkins School of Public Health, Baltimore, MD, USA, a leading public health institution in the United States with a large faculty with diverse research interests. To our knowledge, this is the first survey of its kind.

The Johns Hopkins Bloomberg School of Public Health regularly updates a public listing of its 1564 full- and part-time faculty and researchers. There are 2 primary tracks for faculty—professor or scientist—in addition to positions of varying permanence. We restricted our survey to the 912 faculty in professorial- and scientist-track positions.

We requested all members of an updated faculty email list to complete an anonymous online survey with questions about respondent characteristics, beliefs about social media, and usage of specific technologies, including blogs, Facebook, Twitter, and YouTube. The 30-question survey was administered using a commercial survey website, SurveyMonkey. Three email invitations were sent during a 6-week period in March-April 2011.

We constructed a Social Media Usage Index (SMUI) score with a possible range of 0-16, weighting each social media service equally. For each of the 4 social media services mentioned in the survey (blogs, Facebook, Twitter, and YouTube), respondents were assigned zero points for never having heard of a service, 1 point for having heard of but never using the service, 2 points for using the service but not in the past month, 3 points for using the service once or twice in the past month, and 4 points for using the service 3 or more times in the past month.

Responses were analyzed with Stata statistical software version IC 11.2 (StataCorp LP, College Station, TX, USA). The Johns Hopkins School of Public Health Institutional Review Board reviewed the study and declared it exempt.

## Results

We received 181 usable responses by professor- and scientist-track faculty out of a total of 912 potential respondents in those positions, for a response rate of 19.8%. Unusable responses included incomplete responses and responses by individuals on the faculty email list but not part of the professor- or scientist-track faculty. Respondent characteristics are described in Table 1.

Most respondents rarely used major social media platforms. Respondents were more likely to have used YouTube (94/181, 51.9%) and Facebook (81/181, 44.8%) than to have read blogs (53/181, 29.3%) or used Twitter (12/181, 6.6%) in the prior month. Awareness of these services was nearly universal: only 1 respondent had not heard of Twitter and YouTube, and all respondents had heard of Facebook and blogs. Respondents were much more likely to use all 4 services for personal reasons, but the proportion of use that was predominantly personal for YouTube (163/181, 90.0%) and Facebook (164/181, 90.6%) was greater than for Twitter (146/181, 80.7%) and blogs (149/181, 82.3%). When restricted to respondents who used the services regularly (≥3 times/week) the proportion of mostly personal use was similar for YouTube (160/181, 88.4%), Facebook (165/181, 91.2%), and blogs (122/181, 67.4%); only 6/181 (3.3%) respondents used Twitter this frequently.

Faculty responses seemed to distribute along a continuum of enthusiasm. One respondent, asked about whether faculty should be engaged in social media discussions, replied, “I very rarely look at YouTube, only if someone sends me something. I write 30,000 emails per year. There is no time for additional media of any kind. I am skeptical of researchers who use social media to increase interest in their work.” In contrast, others expressed supportive opinions of engagement, with a caveat of limiting such interaction, “I think it is helpful to be engaged in some social media in the current age; however, maintaining a formal presence takes time most of us don’t have. It is beneficial that my center organizes it for us, and we work with students to do blogging. Unfortunately, credibility as an academic may be affected when blogging too much.”

Finally, a smaller number of faculty recognized that social media is useful to public health professionals “to get information out when trying to build awareness or change policy. The short, informal nature of social media is critical to reaching certain audiences who don’t have the time to weed through long reports or read journal articles.” Another respondent echoed the sentiment, “I think it is very important for raising awareness of a public health issue, for advocacy purposes, and for dissemination of study results.”

**Table 1:** Social Media Usage Index (SMUI) score by respondent characteristics.

Characteristics		Respondents n (%)	SMUI mean (95% CI)^a^
Total		181 (100)	
**Gender**			
	Male	83 (45.9)	8.33 (2.58-14.08)
	Female	97 (53.6)	8.42 (2.94-13.90)
**Age (years)**			
	≤40	49 (27.1)	9.12 (4.07-14.18)
	41-50	47 (26.0)	8.45 (2.91-13.99)
	51-60	43 (23.8)	8.70 (2.83-14.57)
	≥61	42 (23.2)	7.07 (1.83-12.31)
**Years as John Hopkins faculty**			
	0-10	92 (50.8)	8.89 (3.10-14.68)
	11-20	37 (20.4)	8.19 (2.99-13.39)
	≥21	52 (28.7)	7.57 (2.41-12.75)
**Position**			
	Assistant scientist	29 (16.0)	8.66 (3.68-13.63)
	Associate scientist	9 (5.0)	8.78 (2.75-14.80)
	Senior scientist	4 (2.2)	7.50 (2.32-12.68)
	Assistant professor	35 (19.3)	9.03 (3.43-14.62)
	Associate professor	34 (18.8)	7.71 (2.28-13.13)
	Professor	70 (38.7)	8.24 (2.38-14.09)

^a^From bivariate analysis.

**Figure 1:**
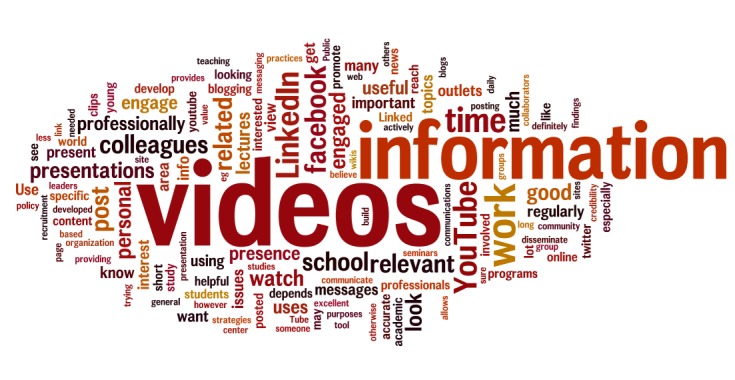
A “Wordle” diagram of survey responses on current and desired uses of social media in public health research and practice.

Social media was seen, in this sample, as more useful for spreading research results than conducting research: 122 (67.4%) agreed that social media is important for disseminating information, whereas only 55 (30.4%) agreed that social media is useful for their research. Respondents were skeptical when asked whether social media engagement was helpful for career advancement; 43 (23.8%) said social media usage was useful, whereas 72 (39.8%) said it was not. A minority were very involved with social media: 43 (23.8%) faculty said they would employ a full- or part-time social media consultant, and 30 (16.6%) currently employed one. Open responses questions indicated considerable enthusiasm for social media from respondents engaged in the field. Figure 1 is a visual diagram (Wordle) that arranges the top 150 most common words in survey respondents’ open response answers, scaling the size of words to match their usage frequency.

We fitted bivariate and multivariate linear regression models with the SMUI score as the dependent variable to identify factors associated with higher social media usage scores (Table 2). Mean SMUI did not differ by gender or age category, but was higher for associate professors. For each additional year since degree completion, the mean SMUI score decreased slightly.

**Table 2:** Correlates of Social Media Usage Index (SMUI) scores in multivariate analysis.

Correlates in model		Difference in mean SMUI score, (95% CI)	*P* value^a^
**Gender**			
	Male	[Reference]	
	Female	0.52 (–0.34, 1.38)	.23
**Age (years)**			
	≤40	[Reference]	
	41-50	–0.59 (–1,83, 0.64)	.35
	51-60	0.19 (–1.34, 1.73)	.81
	≥61	–0.84 (–2.80, 1.11)	.40
**Position**			
	Assistant scientist	[Reference]	
	Associate scientist	0.67 (–2.29, 3.62)	.66
	Senior scientist	0.47 (–0.89, 1.84)	.50
	Assistant professor	0.25 (–1.22, 1.73)	.74
	Associate professor	2.40 (0.68, 4.12)	.006
	Professor	1.73 (–0.43, 3.90)	.12
**Years since degree completion**			
	0 years	[Reference]	
	Per year increase	–0.12 (–0.18, –0.05)	<.001

^a^ *P* values calculated with *t* tests.

## Discussion

Our results suggest that despite a substantial appreciation for the potential of social media to serve as a component of public health strategy, only a small minority of public health faculty are actually engaged in this space professionally. The majority of respondents were either disinterested or actively opposed to professional engagement in the social media space. Although the generalizability of these results is limited somewhat by only soliciting responses at a single institution, the Johns Hopkins Bloomberg School of Public Health is the largest of its type and represents a substantial portion of public health faculty in the United States.

As public discourse on a number of critical health topics continues to be influenced and sometimes shaped by discussions online from Twitter to Facebook, public health faculty should seriously consider when and how public health professionals should engage in these media, but also how personal, institutional, and professional barriers to greater use of social media may be overcome. Strategies for overcoming these barriers may start with greater awareness of specific applications of social media for public health practice and research. Textbox 1 lists social media resources from the Centers for Disease Control and Prevention (CDC), the National Information Center on Health Services Research and Health Care Technology, and the Johns Hopkins School of Public Health. Recognition of engagement in social media also needs to be incorporated into the way faculty are recognized for their professional practice. This, however, will require the need for robust metrics to quantify the reach and impact specific faculty or institutions are having in these spaces. Services like Klout, Tweetlevel, Bloglevel, and Export.ly provide analytics with some interpretive filters which attempt to quantify the influence specific individuals or organizations have on others.

**Social media resources.**

- CDC Social Media page [22]
- CDC’s Health Communicator’s Social Media Toolkit [23]
- Social Media Resources listed by the National Information Center on Health Services Research and Health Care Technology [24]
- Social Media Channels at the Johns Hopkins School of Public Health [25]

In the age of social media, information is no longer constrained to vertical channels of authority; ideas are shared freely between citizens who can inform or misinform the public. As Chou et al [20] pointed out in their 2009 characterization of users of social media for health information, age, socioeconomic status, and ethnicity may be important confounders of access to or use of these channels. It will be important to further explore the degree to which social media information sources play a role in individual decision making, such as whether to vaccinate one’s children or to choose formula over breast-milk. Public health professionals—from physicians to government officials to academic researchers—should strategically adopt new technologies and styles of communication or risk being excluded from this conversation entirely.

To encourage the use of new approaches to information sharing through social media and engagement in public discourse within this space, we call for increased professional discussion of the benefits and risks of more active engagement in social media by public health professionals, both as a means of gathering new information and to influence ongoing discussions of public health importance.

## Multimedia Appendix 1

Survey on faculty social media use.

## Abbreviations

CDC: Centers for Disease Control and Prevention
SMUI: Social Media Usage Index
