# Supplementary material for: Mind the Gap: Social Media Engagement by Public Health Researchers
Source: J Med Internet Res. 2014 Jan 14;16(1):e8. doi: 10.2196/jmir.2982 (PMC3906700; doi:10.2196/jmir.2982)
Supplement: Supplementary file 1 [file jmir_v16i1e8_app1.pdf]

Appendix: Survey on Faculty Social Media Use

1. What is your age?
   a. 25-30
   b. 31-35
   c. 36-40
   d. 41-45
   e. 46-50
   f. 51-55
   g. 56-60
   h. 61+

2. Gender
   a. Male
   b. Female
   c. Prefer not to say

3. What is your current position at the Johns Hopkins School of Public Health (choose all that apply)?
   a. Research Associate / Associate
   b. Assistant Scientist
   b. Senior Scientist
   c. Assistant Professor
   d. Associate Professor
   e. Professor

4. What is your primary department?
   a. Biochemistry and Molecular biology
   b. Biostatistics
   c. Environmental Health Science
   d. Epidemiology
   e. Health, Behavior and Society
   f. Health Policy and Management
   g. International Health
   h. Mental Health
   i. Molecular Microbiology and Immunology
   j. Population, Family, and Reproductive Health

5. How long have you been at Johns Hopkins (as a faculty member)?
   a. ≤5 years
   b. 6-10 years
   c. 11-15 years
   d. 16-20 years
   e. 21-25 years
   f. 26+ years

6. How many years has it been since you earned your terminal degree?

7. Do you currently engage the services of a full-time or part-time social media consultant, or other staff who is actively pursuing a social media strategy for your work? Check Yes or No

8. Do you read, comment, or post on blogs? Check Yes or No

1. I’ve never heard of blogs.
2. I’ve heard of blogs but don’t read them.
3. I’ve read blogs once or twice but didn’t find it particularly useful.
4. I’m a regular reader and subscriber of blogs.

How many hours do you spend using this tool each week?

On the following scale (1-10) please indicate the degree of professional use of this tool, where 10 is completely professional, and 1 is completely personal.

Personal ______________________ Professional
1 2 3 4 5 6 7 8 9 10

Generically, please give examples of how you use the above media for professional purposes.

9. Do you subscribe to RSS feeds? Check Yes or No

1. I’ve never heard of RSS feeds.
2. I’ve heard of RSS feeds but haven’t used them.
3. I’ve read blogs once or twice but didn’t find it particularly useful.
4. I’m a regular user of RSS feeds.

How many hours do you spend using this tool each week?

On the following scale (1-10) please indicate the degree of professional use of this tool, where 10 is completely professional, and 1 is completely personal.

Personal ______________________ Professional
1 2 3 4 5 6 7 8 9 10

Generically, please give examples of how you use the above media for professional purposes.

10. Do you use Facebook? Check Yes or No

1. I’ve never heard of Facebook.
2. I’ve heard of Facebook but haven’t used it.
3. I’ve used Facebook once or twice but didn’t find it particularly useful.
4. I’m a regular user of Facebook.

How many hours do you spend using this tool each week?

On the following scale (1-10) please indicate the degree of professional use of this tool, where 10 is completely professional, and 1 is completely personal.

Personal ———————————— Professional
1 2 3 4 5 6 7 8 9 10

Generically, please give examples of how you use the above media for professional purposes.

11. Do you use Twitter? Check Yes or No

1. I’ve never heard of Twitter.
2. I’ve heard of Twitter but haven’t used it.
3. I’ve used Twitter once or twice but didn’t find it particularly useful.
4. I’m a regular user of Twitter.

How many hours do you spend using this tool each week?

On the following scale (1-10) please indicate the degree of professional use of this tool, where 10 is completely professional, and 1 is completely personal.

Personal ———————————— Professional
1 2 3 4 5 6 7 8 9 10

Generically, please give examples of how you use the above media for professional purposes.

12. Do you use YouTube? Check Yes or No

1. I’ve never heard of YouTube.
2. I’ve heard of YouTube but haven’t used it.
3. I’ve used YouTube once or twice but didn’t find it particularly useful.
4. I’m a regular user of YouTube.

How many hours do you spend using this tool each week?

On the following scale (1-10) please indicate the degree of professional use of this tool, where 10 is completely professional, and 1 is completely personal.

Personal ———————————— Professional
1 2 3 4 5 6 7 8 9 10

Generically, please give examples of how you use the above media for professional purposes.

13. Which of the following best describes your interaction with Collexis, the tool that identifies research interests of other JHU investigators based on publication history and collaborations.

5. I’ve never heard of Collexis.
6. I’ve heard of Collexis but don’t use it.
7. I used Collexis once or twice but didn’t find it particularly useful.
8. I’m a regular user of Collexis.

How many hours do you spend using this tool each week?

On the following scale (1-10) please indicate the degree of professional use of this tool, where 10 is completely professional, and 1 is completely personal.

Personal ________________________ Professional
1 2 3 4 5 6 7 8 9 10

Generically, please give examples of how you use the above media for professional purposes.

14. Are there any other social media tools not mentioned here that you use? Please specify. To what extent is the use professional vs. personal?

15. Please rank the following statements:

	Strongly agree	Agree	No opinion	Disagree	Strongly Disgree
Social media is an important for disseminating public health information, such as research findings or recommendations					
Social media is useful in my research					
Social media can help me to advance in my career					
I can gain valuable public health information using social media sources					
Social media strategies would improve the reach of public health messages to large groups of people					
There is inadequate curation of social media to allow for public health messages to be delivered accurately					
I would hire a social media expert for my research group					

16. Open ended: Feel free to submit your comments on the following questions:

- Please share, briefly, any social media strategies which you are actively engaged in your professional capacity:

- Please describe the extent to which, if any, you believe public health researchers should be engaged professionally in maintaining a presence on any of the above social media outlets:
